# Assessing and Improving Animal Welfare Using Applied Ethology

**DOI:** 10.3390/ani15020213

**Published:** 2025-01-14

**Authors:** Emma Fàbrega, Maria J. Hötzel

**Affiliations:** 1 Animal Welfare Program, Institute for Food and Agriculture Research and Technology (IRTA), Monells, 17121 Girona, Spain; 2 Laboratório de Etologia Aplicada e Bem-Estar Animal, Universidade Federal de Santa Catarina, Rod. Admar Gonzaga 1346, Itacorubi, Florianópolis 88034-001, Brazil

The papers in this Special Issue, entitled “Editorial Board Members’ Collection Series: Behaviour, Applied Ethology and Welfare of Farmed Animals”, bring to the forefront empirical data and theoretical discussions that contribute to the discussion of contemporary issues, from environmental enrichment to improvements in animal welfare, the intensification of animal rearing systems, and innovations like virtual fencing and dietary adjustments to improve animal health and welfare in organic systems. The overarching topic connecting the papers in this Special Issue is the use of behaviour as an indicator in our understanding of and improvement in animal welfare.

The concept of animal welfare has fluctuated over time, following changes in values and beliefs, together with advances in the scientific understanding of animals. A relevant driver in the new definitions of animal welfare has been the higher interest in including affective states, especially positive ones, under a new approach known as Positive Animal Welfare [1]. Indeed, the notion that animal welfare has to do with the subjective experience of individuals underlies most of the attempts to provide operational definitions, from early ones like the so-called Broom’s definition [2] identifying animal welfare as “the state of an animal in relation to its ability to cope with its environment” to more recent conceptualisations linking “animal welfare to the balance of positive and negative experiences over time” [3]. Although it is widely accepted that farm animals are sentient creatures and have been attributed mental states [4], understanding and assessing their subjective experiences constitute a challenge. Along that line, applied ethology, the study of behaviour, has become a cornerstone to provide useful insights in what animal welfare is and how to assess and improve it.

Unravelling behaviour has been a crucial aim for philosophers and scientists from ancient times, since Aristotle. Charles Darwin was appointed as the great grandfather of ethology [5]. The concept and scope of ethology have also evolved over time. In the first half of the 20th century, the study of animal behaviour became a quantitative scientific discipline, which aimed to work out the physiological causation, ontogeny, function, and evolution of behavioural traits. Konrad Lorenz’s work [6] evaluating the causality of behaviour as a result of the interaction of external stimuli and innate, preprogrammed action patterns has been widely cited. Niko Tinbergen’s “Four Questions” (i.e., “Function”; “Causation”; “Ontogeny”; and “Phylogeny” of Behaviour) [7] today still serve as the undisputed guideline for the study of behaviour [5]. Later on, in the 1960s, the new concept of behavioural ecology emerged as a subdiscipline focusing on the function of behaviour, and at the turn of the 21st century, “behavioural biology” appeared, encompassing a prolific variety of subdisciplines and serving as basis for many other scientific assessments, like animal welfare. It has been argued that out of the various disciplines in agriculture and veterinary sciences, applied ethology could be the one that is more closely associated with animal welfare [8].

The Brambell report [9] was one of the initial influential opinions in identifying applied ethology as the discipline best able to address issues raised concerning modern production methods. The framework of the “Five Freedoms” focused mostly on absence or freedom from negative experiences (i.e., freedom from hunger and thirst, from discomfort, from pain/injury or disease, to expressing normal behaviour and from fear and distress). Over time, the Five Freedoms Framework developed into the “Five Domains” (Nutrition, Physical environment, Health, Behavioural interactions, Mental state) [10], encompassing positive and negative affective states, in an attempt to recognize that animal welfare goes beyond the avoidance of negative experiences and states and needs to promote the achievement of positive experiences. The role of applied ethology in the assessment of animal welfare in this new Five Domains paradigm may be even more crucial. Applied ethologists have been at the forefront of the development of animal welfare science, using a “whole animal” approach to provide information [11]. At a scientific fundamental level, applied ethologists investigated the causation and development of behavioural systems, thus providing some scientific basis for the notion of ’behavioural needs’ [12,13]. For example, early work at the Pig Park, a wooded hill enclosure to create a semi-natural environment for pigs, identified key behavioural patterns in order to apply knowledge to improve welfare in farming systems [14]. Behaviour has also been used to understand the stress response [15,16], and a causal link between behaviour and health has been proposed [17]. At a more applied level, the impact of the design of housing and equipment [18] and management practices [19] on behaviour has long been investigated to better meet the needs of animals and enhance their welfare. The more recent approach of including affective states as an essential part in the welfare status of the individual has given rise to the development of behavioural tests to better understand animal emotion [20,21,22]. Overall, the importance of behaviour and applied ethology to understand, assess, and improve animal welfare has increased over time and expanded to new disciplines of knowledge. This Special Issue is a collection of papers encompassing different species and targeting different aims in which behaviours are a key element in the assessment of or improvement in animal welfare, including both the avoidance of negative experiences and promotion of positive affective states. As proposed in the objectives of the Special Issue, these papers collectively highlight the study of behaviour as an indicator of welfare in farmed animals, and the influence of genetic selection, environmental management, and housing conditions on the welfare of animals.

A group of studies focused on enrichment as a tool to enhance animal welfare through both experimental and conceptual approaches. Two experimental studies examined how specific enrichments—like environmental complexity, sensory stimuli, and object play—promote positive behaviours and reduce stress in animals. Evans et al. [23] explored spatial and behavioural enrichment in broilers, while Lima et al. [24] examined the impact of object play on spotted pacas (*Cuniculus paca*). Evans et al. [23] examined how environmental complexity and stocking density impact the welfare of broiler chickens, particularly through behavioural outcomes. The research used both high-complexity (HC) and low-complexity (LC) environments across varying stocking densities, concluding that HC environments, with spatial diversity and enriched areas for specific activities, enhance behaviours considered to be indicators of positive welfare, like foraging and preening. While dustbathing and play behaviours remained unaffected, age and density influenced activity and behavioural expressions, suggesting that this type of enrichment might reduce stress, though it may also lose efficacy as birds mature. Lima et al. [24] investigated object play as a potential indicator of positive emotional states in farmed spotted pacas, using boomer balls to stimulate behaviours and assess their association with welfare markers like affiliative behaviours and low-amplitude vocalizations using an ABA experimental design. Significant increases in affiliative and exploratory behaviours and a reduction in agonistic interactions followed the introduction of boomer balls, suggesting enhanced welfare conditions when the animals were exposed to these objects. This study confirmed the potential of object play to enhance welfare in captive settings, especially by encouraging more naturalistic, positive behaviours that align with play’s theoretical role in emotional well-being. Additionally, it provided evidence that object play can be a non-invasive measure of positive affective states in this species. Mota-Rojas et al. [25] covered tactile and auditory enrichment for dairy cattle, systematically reviewing recent studies on these stimuli, focusing on mechanical brushes, music, and visual aids like mirrors and images of conspecifics. Considering each method’s benefits in relation to natural behaviour stimulation and stress reduction, the authors concluded that sensory enrichment is a practical approach to enhance positive affective states in dairy cows. Zapata-Cardona et al. [26] propose that music could function as emotional enrichment across species, grounding their insights in comparative biology and psychology. This paper analyses the biological foundations of emotional responses to music across species, suggesting that emotional experiences are not unique to humans but rather embedded in shared neural mechanisms. This perspective is innovative, challenging traditional views that restrict music and emotion to a human context, and it posits that animals may similarly benefit from music, which could improve their welfare. Together, this group of studies emphasize the role of environmental enrichment in facilitating natural behaviours or reducing stress in captive animals to improve welfare.

Two studies in this SI emphasize the role of innovations like virtual fencing and dietary adjustments to improve livestock health and environmental management. Bagaria et al. [27] investigated dietary strategies to prevent post-weaning diarrhoea in organic piglets through variations in crude protein levels and whey supplementation. Although the experimental diets (high protein, low protein, and low protein supplemented with liquid whey) had no significant impact on post-weaning diarrhoea incidence, piglets receiving whey supplementation showed higher average daily gain, reduced frequency of negative social behaviours, and beneficial changes in their intestinal microbiota. This suggests that while dietary protein reduction alone may be insufficient for post-weaning diarrhoea prevention in organic settings, whey supplementation could offer growth and health benefits, aligning with organic farming standards. Aaser et al. [28] examined the use of Global Navigation Satellite System—GNSS—and virtual fencing to monitor cattle habitat preferences for grazing, resting, and ruminating behaviours, showcasing remote monitoring’s potential for welfare and environmental management by reducing the overuse of resources and monitoring and detecting health and welfare issues of cattle rapidly.

Conducted on 16 commercial laying hen farms in Germany, the study by Hüttner et al. [29] explored fear responses, assessed through a novel object test. Increased fear responses were associated with higher occurrences of feather damage and cannibalism. Furthermore, fear responses, which increased with age and flock size, were influenced by genetics and were more pronounced in hens in barn systems than in free-range systems, hinting at the potential welfare benefits of outdoor access in reducing stress-related behaviours.

Phillips [30] and Nogueira and Hötzel [31] focused on philosophical and socio-economic dimensions of animal welfare and applied ethology. Phillips [30] provides a conceptual analysis of animal welfare from farmers’ perspectives, addressing how external pressures like climate change, labour shortage, and economic constraints shape animal welfare practices. This article also discusses the feasibility and limitations of strategies that may help farmers address these welfare challenges, such as improved nutrition, diversification, silvopastoral systems, genetic adjustments, and precision livestock farming technologies. For example, he argues that while automation may help in part the shortage of skilled workers with an ability to detect animal behaviours, cost, effectiveness, and negative farmer perception may limit the uptake of these technologies. Nogueira and Hötzel [31] examined how farmers’ perceptions shift as they move from pasture-based to more intensive, confined dairy systems. This study, set in southern Brazil, an area where dairy farming is undergoing intensification, highlights the contrasting views of farmers maintaining pasture-based systems and those utilizing or transitioning to confined systems. The first often emphasize the importance of natural living conditions and natural behaviours; the latter, in contrast, associated cow welfare more closely with factors like comfort and health management within a confined setting. Concepts such as resilience, longevity, and affective states were interpreted differently depending on the farming context and farmers’ values. This duality highlights how the intensification of dairy farming can change welfare conceptions, positioning comfort and productivity as primary welfare indicators in confined systems. Both articles [30,31] highlight the complexities involved in aligning animal welfare with economic realities and indicate the need for policies that cater to different farm sizes and address the balance between welfare and economic constraints.

Overall, positive emotions and positive welfare are discussed in many papers of this Special Issue [23,24,25,26,31], highlighting the growing interest in improving animal welfare by providing animals opportunities to enjoy positive welfare rather than simply avoiding pain and suffering. All nine articles share a commitment to advancing animal welfare by exploring innovative ways to assess and improve the well-being of farmed and captive animals based on the use of applied ethology. Each study examined specific strategies—technological, behavioural, or environmental—that may be used to enhance animals’ living conditions and reduce stress. Whether through enrichment methods (like music and object play), technological tools (such as virtual fencing), or examining farmers’ evolving perceptions of welfare, each article recognizes the influence of external pressures (economic, environmental, or technological) on the implementation of welfare practices.
